# Radical-Driven Methane Formation in Humans Evidenced by Exogenous Isotope-Labeled DMSO and Methionine

**DOI:** 10.3390/antiox12071381

**Published:** 2023-07-04

**Authors:** Frank Keppler, Mihály Boros, Daniela Polag

**Affiliations:** 1 Institute of Earth Sciences, Heidelberg University, D-69120 Heidelberg, Germany; daniela.polag@geow.uni-heidelberg.de; 2 Heidelberg Center for the Environment (HCE), Heidelberg University, D-69120 Heidelberg, Germany; 3 Institute of Surgical Research, University of Szeged, H-6724 Szeged, Hungary; boros.mihaly@med.u-szeged.hu

**Keywords:** endogenously produced methane, reactive oxygen species, methyl radicals, dimethyl sulfoxide, methyl donors, stable carbon and hydrogen isotope labeling, oxidative stress, antioxidant defense system

## Abstract

Methane (CH_4_), which is produced endogenously in animals and plants, was recently suggested to play a role in cellular physiology, potentially influencing the signaling pathways and regulatory mechanisms involved in nitrosative and oxidative stress responses. In addition, it was proposed that the supplementation of CH_4_ to organisms may be beneficial for the treatment of several diseases, including ischemia, reperfusion injury, and inflammation. However, it is still unclear whether and how CH_4_ is produced in mammalian cells without the help of microorganisms, and how CH_4_ might be involved in physiological processes in humans. In this study, we produced the first evidence of the principle that CH_4_ is formed non-microbially in the human body by applying isotopically labeled methylated sulfur compounds, such as dimethyl sulfoxide (DMSO) and methionine, as carbon precursors to confirm cellular CH_4_ formation. A volunteer applied isotopically labeled (^2^H and ^13^C) DMSO on the skin, orally, and to blood samples. The monitoring of stable isotope values of CH_4_ convincingly showed the conversion of the methyl groups, as isotopically labeled CH_4_ was formed during all experiments. Based on these results, we considered several hypotheses about endogenously formed CH_4_ in humans, including physiological aspects and stress responses involving reactive oxygen species (ROS). While further and broader validation studies are needed, the results may unambiguously serve as a proof of concept for the endogenous formation of CH_4_ in humans via a radical-driven process. Furthermore, these results might encourage follow-up studies to decipher the potential physiological role of CH_4_ and its bioactivity in humans in more detail. Of particular importance is the potential to monitor CH_4_ as an oxidative stress biomarker if the observed large variability of CH_4_ in breath air is an indicator of physiological stress responses and immune reactions. Finally, the potential role of DMSO as a radical scavenger to counteract oxidative stress caused by ROS might be considered in the health sciences. DMSO has already been investigated for many years, but its potential positive role in medical use remains highly uncertain.

## 1. Introduction

Methane (CH_4_) is an important and highly abundant carbon molecule in the Earth’s atmosphere that affects the Earth’s radiative balance. Around 600 to 700 million tons of CH_4_ are released into the atmosphere annually by natural and anthropogenic sources, mostly of biological origin [1]. For a long time, biological CH_4_ formation was considered to only occur from the metabolism of microorganisms—methanogens that belong to the domain Archaea—living under strictly anaerobic conditions in natural wetlands; landfills; rice fields; or in the alimentary tract of vertebrates, including ruminants and humans. However, in 2006, it was clearly demonstrated that plants—from the Eukarya domain—are able to produce CH_4_ per se [2]. This breakthrough finding fueled research to search for alternative biological CH_4_ sources other than those derived from archaeal methanogens. Since then, many studies unambiguously confirmed direct (endogenous) CH_4_ formation and release from eukaryotes, including plants [3,4,5,6,7,8], animals [9,10], fungi [11,12], and marine and freshwater algae [13,14,15]. In addition, cyanobacteria—belonging to the domain Bacteria—thriving in aquatic and terrestrial environments are also able to generate CH_4_ at substantial rates depending on the species and environmental conditions [16]. Finally, a universal formation mechanism for CH_4_ was recently proposed that might explain why all living organisms produce CH_4_ under aerobic conditions [17]. In conclusion, the recent findings have stimulated great interest in endogenous CH_4_ formation processes all over the three life domains, which are now often referred to as “aerobic”, “non-archaeal”, or “non-microbial” CH_4_ formation processes [18]. Therefore, in the following paragraphs, we first discuss the traditional view of CH_4_ formation before we deal with the recently identified alternative pathways.

### 1.1. Traditional View of Microbial Methane Formation in Humans

Traditionally, CH_4_ formation in humans was considered to exclusively arise from anaerobic microbial activity in the gastrointestinal tract [19,20,21]. The microbial species identified so far in the distal part of the colon (methylotrophic *Methanospaera stadtmanae* and hydrogenotrophic *Methanobrevibacter smithii*) were considered to contribute to the observed 25% to 70% of humans defined as CH_4_ producers. The terms “CH_4_ producer” (with breath CH_4_ emission > 1 part per million (ppmv) above background values) and “CH_4_ non-producer” (breath CH_4_ emission < 1 ppmv above background values) used in many previous publications have become somewhat misleading after Keppler et al. [22], as demonstrated through high-precision measurements that showed that every human produces at least 26 parts per billion by volume (ppbv) breath CH_4_ above background CH_4_ values. This value is around three orders of magnitude lower when compared with the average values from typical breath CH_4_ “producers”. Based on these results, the following preferred terminology was proposed: high (>4 ppmv), medium (1–4 ppmv), and low (<1 ppmv) breath CH_4_ producers instead of CH_4_ producers and non-producers.

Regarding the CH_4_-producing status, several studies suggested that factors such as age [23,24], ethnic background [25,26], gender [24,27], exercise status [28], and various gastrointestinal diseases [29,30,31,32,33,34] play roles in an increased level of breath CH_4_. Polag and Keppler [35] gave an overview of the variability of study parameters and calculated that 38% of humans globally are high producers of CH_4_, with an average CH_4_ content of around 17 ppmv in breath air. For further discussion regarding the microbial formation of CH_4_ in the human gut system and the physiological factors that might control these processes, see the review articles by de Lacy Castello et al. [36] and Levitt et al. [20].

Hereafter, based on the stable carbon isotope and CH_4_ emission patterns of various age groups, it was hypothesized that next to microbial sources in the gastrointestinal tracts, there might be other, yet unidentified, endogenous cellular processes involved in CH_4_ formation [18,22,37]. Indeed, long-term monitoring of CH_4_ in combination with the observation of physical conditions suggested a relationship between deviations in breath CH_4_ production from the base level and immune reactions and inflammatory processes [37,38]. Thus, there is increasing evidence that CH_4_ has a bioactive role in the cellular physiology of eukaryotes and could be considered a diagnostic marker of oxido-reductive stress [18,39].

### 1.2. Alternative Mechanism(s) of Non-Microbial Methane Formation in Eukaryotes

In plants, it was shown that CH_4_ production is influenced by environmental factors, such as visible light [4], ultraviolet-B radiation [5,6,40,41,42,43,44], and temperature [2,6,42,45]. Several precursors of non-microbial CH_4_ production, including methoxy groups of plant pectins [2,5,46], lignin, cellulose [45], ascorbic acid [47], L-methionine [8,48], and epicuticular wax [49], were suggested. In addition, for higher plants and microalgae, it was demonstrated that environmental stressors drastically enhance CH_4_ formation [14,50].

To understand non-microbial CH_4_ formation in eukaryotes and other organisms, it is important to consider the role of reactive oxygen species (ROS, e.g., hydroxyl radicals (•OH), superoxide radicals (O_2_*^−•^*), hydrogen peroxide (H_2_O_2_), or carbonate radicals (CO_3_*^−•^*)), iron species, and carbon precursor compounds. In highly oxidative environments generated in vitro by a chemical model system containing iron (II/III), H_2_O_2_, and the radical scavenger ascorbate, CH_4_ is readily formed from organosulfur and nitrogen compounds, with the highest conversion rates found for DMSO [51]. Under these Fenton-type conditions, in the presence of H_2_O_2_, nonheme oxo-iron(IV) ([Fe^IV^=O]^2+^) oxidizes methyl sulfides to sulfoxides, which then results in the selective formation of methyl radicals via sulfoxide demethylation and ultimately leads to CH_4_ [51,52]. Alternatively, ROS can directly react with methyl sulfides to produce methyl radicals or peroxomethyl radicals in the presence of oxygen [53,54,55], subsequently resulting in CH_4_ formation or oxidized C1 species, such as methanol or formaldehyde.

The traditional homogenous Fenton reaction includes the interaction of free iron species and H_2_O_2_ (Equation (1)). This is a key reaction in biological systems and its major cause is oxidative stress. For detailed information regarding the various reaction steps of the Fenton process, we refer to the literature [56,57]. In living cells, Fenton chemistry takes place, as iron is an essential trace element [58] and H_2_O_2_ is produced during respiratory, and generally metabolic, activity [59]. Hydrogen peroxide and ferrous iron (Fe^2+^) either react to give ferric iron (Fe^3+^), OH^−^, and •OH (Equation (1)), or [Fe^IV^=O]^2+^ and water [60]. This provides the basis for our understanding of CH_4_ formation in cells under oxic conditions. A wide spectrum of molecules that act as methyl donors for CH_4_ formation are conceivable. However, of particular interest are compounds with sulfur (S)- and nitrogen (N)-bonded methyl groups that arise during cellular metabolism or are externally provided. For methylated sulfur compounds, these include DMSO and methionine, which are ubiquitous in the environment [61]. In addition, methylated nitrogen compounds, such as betaine, choline, or trimethylamine, might also serve as CH_4_ precursors. This was recently shown for many organisms from the three domains of life using culture experiments, and a detailed reaction mechanism for CH_4_ formation was suggested, highlighting the interaction between ROS, iron, and S- and N-methylated compounds [17]. The authors also showed that oxidative stress led to increased CH_4_ formation in the studied organisms.

Fe(II) + H_2_O_2_ → Fe(III) + OH^−^ + •OH (1)

In summary, the reaction of methylated sulfur compounds, such as DMSO and methionine with Fenton-type chemistry involving ROS, carbonate radicals, or oxo-iron(IV) results in the formation of methyl radicals (•CH_3_), of which a portion reacts to CH_4_ through abstraction of a hydrogen atom from hydrocarbons, hydrogen peroxide, or hydrogen carbonate. Alternatively, the methyl radicals form oxidized C1 species, such as methanol, formaldehyde, or formic acid. Thus, it is conceivable that there is in vivo formation of C1 compounds as a result of ROS formation and interaction with methylated compounds. Therefore, we consider the administration of isotopically labeled DMSO and methionine as ideal model compounds to confirm the occurrence of ROS-driven CH_4_ formation in humans.

### 1.3. Application of DMSO to Humans

Dimethyl sulfoxide (DMSO) is an organic polar aprotic molecule that was first synthesized in 1866. It was used as an important solvent for many decades before being proposed for use as a pharmaceutical in the 1960s by Stanley Jacob. Because of its ability to rapidly penetrate through human skin and its properties as a free radical (•OH) scavenger, it was widely used as an anti-inflammatory, antipain, and neuroprotective agent. A wide range of biological and pharmacological effects of DMSO were described by Jacob and Herschler [62] for the interested reader. Since 1978, DMSO has been approved by the United States Food and Drug Administration (FDA) for the treatment of interstitial cystitis. Other medical applications, as well as potential physiological and pathological effects of DMSO, are highly controversially discussed. For example, Amemori et al. [63] found that the oral administration of DMSO is an effective treatment for amyloid A amyloidosis. On the other hand, experiments with rats found that DMSO might induce retinal apoptosis [64]. Despite the differing results of the various studies, it is generally assumed that DMSO is nontoxic below 10% (*v*/*v*) [65] with an oral medium lethal dose of 28,300 mg/kg (rat) and a dermal medium lethal dose of 40,000 mg/kg (rat).

### 1.4. Aims and Postulates

Recent results showed that CH_4_ might be formed in all organisms and that the formation of methyl radicals induced by ROS is a prerequisite for the generation of CH_4_. The experiments described in this paper were undertaken in order to unambiguously demonstrate (as a first proof of principle) that CH_4_ is endogenously formed in humans via a radical-driven process without the involvement of the well-known microbial sources (methanogens) living in the gastrointestinal tract. Therefore, a volunteer—the first author of this study—applied isotopically labeled (^2^H or ^13^C) DMSO on the skin (arm), consumed it via the mouth, and applied it to blood samples. In addition, the amino acid methionine (with an isotopically labeled ^13^C methyl group) was also applied to the blood samples. The released gases were analyzed for their isotopic composition to unambiguously identify the formation of CH_4_ from the precursor compounds DMSO and methionine. Based on the results and the formation patterns observed, we discuss several hypotheses concerning the origin of cell-based CH_4_ production and its potential physiological role in mammals. Finally, as DMSO has already been investigated for many years while its potential positive role for medical use is highly uncertain, we briefly discuss the potential application of DMSO to reveal and counteract oxidative stress.

## 2. Materials and Methods

### 2.1. Subject, Materials, Experiments, and Sampling of Air

#### 2.1.1. Subject of the Study

All experiments and measurements were conducted by the principal investigator (PI) and first author of this study (F.K.) from June 2018 to October 2020. The subject was a healthy 55-year-old man without known disease, prescribed medications, or drug intake. The average breath CH_4_ production value of the subject was 9 ± 6.7 ppmv, measured over 72 weeks [38], and he was thus classified as a medium-to-high emitter (see the explanation above). Air and blood samples were provided by the PI, as shown in Section 2.1.3 below. A surrogate investigator (D.P.) was designated to obtain informed consent from the self-experimenter (F.K.), in agreement with the ethics relevant to solitary self-experimentation [66]. The work described was carried out in accordance with The Code of Ethics of the World Medical Association. The research was reviewed by the Medical Research Council of Hungary (ETT-TUKEB) and it was approved as part of the protocol “Mapping metabolic pathways of endogenous gas formation by isotopic analysis of the gas composition of human samples” (6420-8-2023/EUIG/768).

#### 2.1.2. Materials: Position-Specific Isotopically Labeled DMSO and Methionine

**Figure 1:**
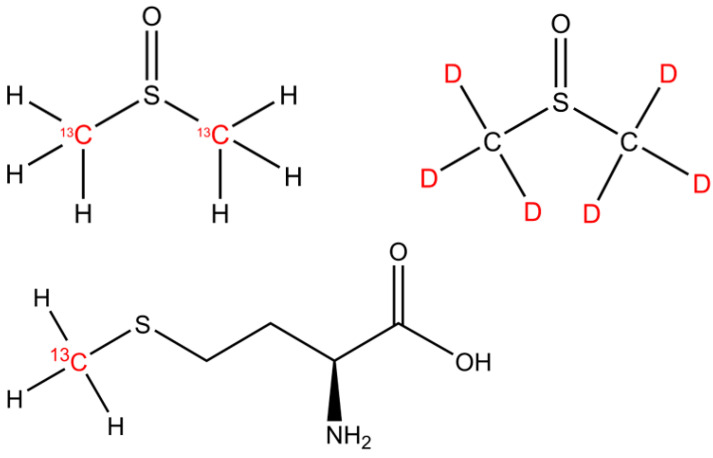
The chemical structures of DMSO and methionine. The isotopically labeled carbon and hydrogen positions are indicated by the ^13^C and D (deuterium, ^2^H) highlighted in red font.

The isotopically labeled sulfur-bonded methyl group(s) in DMSO (^13^C2-DMSO, 99%, Campro Scientific GmbH, Berlin, Germany and DMSO-d6, 99.9 atom %; Sigma Aldrich, Taufkirchen, Germany) and methionine (^13^CH_3_-MET, Sigma-Aldrich, Taufkirchen, Germany; Isotec 99% ^13^C atoms) were investigated as methyl precursors for CH_4_ (Figure 1). Please note that ^2^H-labeled methionine was not available for the experiments.

#### 2.1.3. Experiments and Sampling of Air

**Figure 2:**
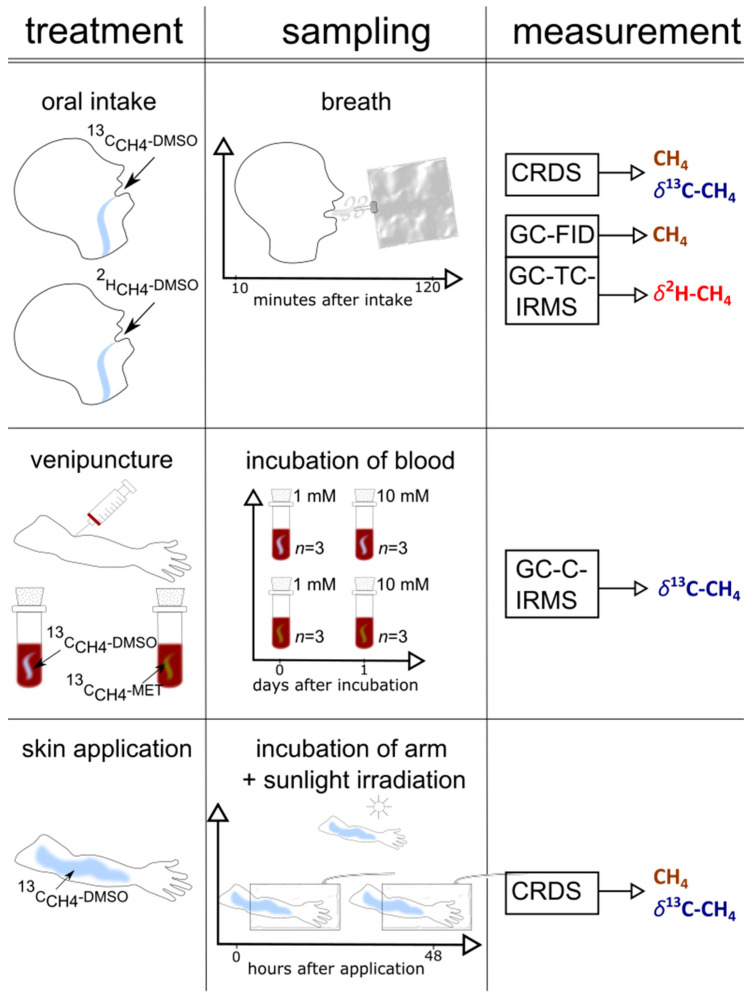
A graphical representation of the set-up (treatment, sampling, measurement) of the three experiments (skin, blood, and oral administration) performed in this study.

A graphical representation of the set-up of the three individual experimental series (oral intake, arm exposure to sunlight, and blood experiments), including the collection of samples and the applied measurements, is outlined in Figure 2. Table S1 shows the timeline of the experiments.

#### 2.1.4. Oral Intake of ^13^C- and ^2^H-Labeled DMSO

The volunteer of the study swallowed 100 µL ^13^C-CH_4_ DMSO (4% ^13^C-content, dissolved in 300 mL H_2_O) or 1 mL of ^2^H-CH_4_ DMSO (10% ^2^H-content, dissolved in 300 mL H_2_O), respectively. Subsequently, the breath CH_4_ concentration and isotope values of CH_4_ (*δ*^13^C or *δ*^2^H, respectively) were monitored for 130 min. The breath samples were collected using 1 L Tedlar bags. The breath CH_4_ sampling procedure was performed in a consistent manner. During the breath air collection, the volunteer breathed normally, stopped breathing for around 5 s, and then filled the Tedlar bag with expired air (range of 0.8 to 1 L). Depending on the study parameter, the gaseous sample was analyzed using cavity ringdown spectroscopy (CRDS), gas chromatography flame ionization detection (GC-FID), or gas chromatography temperature conversion isotope ratio mass spectrometry (GC-TC-IRMS) immediately after sampling (see analytical measurements below).

#### 2.1.5. Arm Incubations and Exposure to Solar Light

For the CH_4_ skin emission analysis, the forearm of the subject was placed inside a cylindrical chamber (see photo documentation 1 in the Supplementary Materials) made of polytetrafluorethylene (PTFE) foil (transparent for UV light), with a diameter of 18.5 cm and a length of 42.5 cm (volume = 11.7 L). The round opening at the back was sealed with an elastic PTFE foil tied to the chamber and fixed along the upper arm. A gas inlet and outlet PTFE tube system was attached to the chamber. The pressure of the chamber was constant during the whole monitoring phase. Ventilation at the inside front of the chamber provided a homogeneous air mixture. The outlet tube was directly connected to the CRDS system (see analytical measurements below) for in situ online analysis of CH_4_ and CO_2_ concentrations and *δ*^13^C values. First, the empty chamber (filled with laboratory air) was measured as a control. Next, the volunteer thoroughly washed his arm with tap water and dried it with a paper towel before placing it in the chamber for 30 min to obtain a control value. Then, ^13^C-CH_4_-labeled DMSO (a mixture of 400 µL DMS0 + 100 µL ^13^C-labeled DMSO + 500 µL H_2_O) was thoroughly distributed on the skin of the left upper forearm (penetrated area of around 30 cm^2^) and the air in the chamber was connected to the CRDS measurement system for a monitoring period of 1 h. Afterward, the forearm was exposed to natural solar light in the field for a period of 1 h (from 10 to 11 am, in July in Heidelberg, Germany). After returning from the field to the laboratory (within 5 min) the left arm was again placed in the chamber and monitored for changes in the *δ*^13^C-CH_4_ values for 1 h. The same procedure was repeated the following two days and the untreated right arm served to record control values.

#### 2.1.6. Blood Samples and Incubation with DMSO and Methionine

Approximately 20 mL of whole-blood samples were collected from the PI through venipuncture by using 4 × 7.5 mL S-Monovettes^®^ containing Ethylenediaminetetraacetic acid (EDTA) to prevent coagulation. Samples were immediately processed for isotope label experiments. Therefore, ^13^C-labeled DMSO and methionine were added to 1 mL of blood in autoclaved 40 mL headspace vials (Supelco 27184) so that the final concentration of the added compound was 1 mM or 10 mM. Vials were sealed using a hole-type screw cap (Supelco) fitted with a PTFE/silicone septum (Supelco). The control samples were prepared in the same way, except that the added DMSO and methionine were isotopically not enriched in ^13^C. All samples were prepared in triplicates and incubated at 36 °C for 24 h before the gas phase in the vials was analyzed (first day). Afterward, the vials were opened to equilibrate with the air in a fume cupboard. After 30 min, the samples were again sealed with a PTFE/silicone septum and incubated at 36 °C for 24 h before the gas phase was analyzed (second day).

### 2.2. Analytical Measurements

The analytical laser technique applied in this study to obtain online stable carbon isotope measurements and concentrations of CH_4_ was almost identical to that described previously [22]. In addition, stable carbon and hydrogen isotope analyses were conducted by applying GC-IRMS, as described in Einzmann et al. [67]. However, we briefly describe the applied analytical techniques in the sections below. For more analytical details, and the application of stable isotope techniques, please refer to the studies by Keppler et al. [22] and Einzmann et al. [67] and to the Supplementary Materials.

#### 2.2.1. Natural Abundance of ^13^C/^12^C and ^2^H/^1^H, Definition of *δ* Values, Isotopic Excess, and Keeling Method

Throughout this paper, the “delta” (δ) notation—which is the relative difference of the isotope ratio of a material to that of a standard V-PDB (Vienna Pee Dee Belemnite, ^13^C/^12^C ratio of 0.011108) or V-SMOW (Vienna Standard Mean Ocean Water, ^2^H/^1^H ratio of 0.00011576)—is used; values of *δ*^13^C and *δ*^2^H relative to those of V-PDB and V-SMOW, respectively, are defined by the following equations:

*δ*^13^C = ((^13^C/^12^C)_sample_/(^13^C/^12^C)_standard_) − 1. (2)

*δ*^2^H = ((^2^H/^1^H)_sample_/(^2^H/^1^H)_standard_) − 1. (3)

To comply with the guidelines of the International System of Units (SI), we followed the proposal of Brand and Coplen [68] and used the term urey, after H.C. Urey (symbol Ur), as the isotope delta value unit. In such a manner, an isotope-delta value expressed traditionally as −60‰ can be written as 60 mUr. For natural sources of CH_4_, typical *δ*^13^C-CH_4_ and *δ*^2^H-CH_4_ values are in the range of −20 to −100 mUr [12] and −100 to −400 mUr [44], respectively.

The isotopic difference (Δ) between the control and sample is defined as

Δ = *δ*^13^C_sample_ − *δ*^13^C_control_ (4)

The ^13^C % and ^2^H % excesses were calculated as follows:

(5) C13 %excess=C13C13+C12Labelled−C13C13+C12Basis∗100=C13 %Labelled−C13 %Basis

(6) H2 % excess=H2H2+H1Labelled−H2H2+H1Basis∗100=H2 %Labelled−H2 %Basis

#### 2.2.2. Laser Absorption Spectroscopy—Cavity Ringdown Spectroscopy

##### Measurements of CH_4_ Concentrations and Stable Carbon Isotope Values

Cavity ringdown spectroscopy is a highly sensitive optical spectroscopic technique for the measurements of both the stable carbon isotope value (*δ*^13^C-CH_4_) and the concentration of CH_4_. The Tedlar gas sample bag (from breath air) or the arm incubation Teflon chamber (see the Supplementary Materials) was connected to the CRDS, and the flow rate to the analyzer was 23 mL/min. Before entering the analytical system, the gas was passed through two chemical traps filled with AscariteII^®^ (sodium hydroxide coated silica) and Drierite^®^ (anhydrous CaSO_4_) to remove the carbon dioxide (CO_2_) and water, respectively. This was necessary due to the higher concentrations of CO_2_ and water (up to 6%) in the breath sample, which can cause interferences with the spectroscopic CH_4_ measurements.

Stable carbon isotope values and concentrations of CH_4_ were measured with a G2201-i cw-CRDS-Analyzer (Picarro, Inc., Santa Clara, CA, USA). This instrument enables simultaneous measurements of the CH_4_ concentration, *δ*^13^C-CH_4_ value, and water content in a gas sample. The concentration precision (1σ, 2 min average) specified by the manufacturer was 50 ppbv + 0.05% of reading (^12^C) and 10 ppbv + 0.05% of reading (^13^C) in the high dynamic range mode, and 5 ppbv + 0.05% of reading (^12^C) and 1 ppbv + 0.05% of reading (^13^C) in the high precision mode. The *δ*^13^C-CH_4_ precision provided by the manufacturer was <0.8 mUr. However, typical standard deviations (SD) of measurements of breath samples and standards (using filled Tedlar bags) were in the ranges of ±1.2 ppbv and ±0.3 mUr (1σ, 2 min average measurement interval) for the concentration and stable isotope measurements, respectively (see also [22]).

In order to quality assure the *δ*^13^C-CH_4_ values, some gas samples were measured using both CDRS and gas chromatography–combustion–isotope ratio mass spectrometry (GC-C-IRMS) (for details, see Section 2.2.4. below). Samples measured via IRMS were analyzed three times (*n* = 3) and the average standard deviations of the analytical measurements were in the range of 0.1 to 0.3 mUr. The measured difference between the two analytical systems was used to normalize the isotope data of the CRDS.

#### 2.2.3. Measurements of CH_4_ Concentrations Using Gas Chromatography Flame Ionization Detection

An aliquot (5 mL) of headspace gas was taken from the incubation vials (40 mL) or gas bags (1 L) using a gastight syringe. Before entering the analytical system, the gas sample was passed through a chemical trap filled with Drierite^®^ to remove water. The sample gas was separated via gas chromatography using a GC-14B (Shimadzu, Japan) equipped with a 2 m column (Ø = 3.175 mm inner diameter) packed with a Molecular Sieve 5A 60/80 mesh from Supelco. Methane was recorded using an FID, and its concentration was quantified by using two reference gases containing 9837 ppbv and 2192 ppbv CH_4_.

#### 2.2.4. Continuous Flow Isotope Ratio Mass Spectrometry

##### Measurement of *δ*^13^C-CH_4_ Values

Gas from the Tedlar gas bags (from breath samples) or 40 mL glass vials (headspace of blood samples) was transferred to an evacuated sample loop (40 mL). Interfering compounds were separated via GC and CH_4_ was trapped on Hayesep D. Afterward, CH_4_ was separated from the interfering compounds via GC and transferred to a gas chromatography–combustion–isotope ratio mass spectrometer (Deltaplus XL mass spectrometer, ThermoQuest Finnigan, Bremen, Germany) via an open split. The working reference gas was CO_2_ of high purity (carbon dioxide 4.5, Messer Griesheim, Frankfurt, Germany) with a known *δ*^13^C value of −23.64 mUr (calibrated at MPI for Biogeochemistry in Jena, Germany). All *δ*^13^C-CH_4_ values were corrected using two CH_4_ working standards (isometric instruments, Victoria, BC, Canada) calibrated against the National Institute of Standards and Technology (NIST) and International Atomic Energy Agency (IAEA) reference substances. The calibrated *δ*^13^C-CH_4_ values of the two working standards in mUr vs. V-PDB were −23.9 ± 0.2 and −54.5 ± 0.2. All samples were normalized via two-scale anchor calibration according to Paul et al. (2007).

##### Measurement of *δ*^2^H-CH_4_ Values

δ^2^H-CH_4_ values were determined via GC-TC-IRMS. The same analytical set-up was applied as for stable carbon isotope measurements (see Section 2.2.2 above) with the following modifications: The flow rate was 0.6 mL min^−1^ and instead of combustion to CO_2_ and H_2_O, CH_4_ was thermolytically converted (at 1450 °C) to produce hydrogen (H_2_) and carbon. After IRMS measurements of the hydrogen, the obtained *δ*^2^H values were normalized using two reference standards of high-purity CH_4_ with δ^2^H values of—190.6 ± 0.2‰ (in-house) and—149.9 ± 0.2‰ (T-iso2, Isometric Instruments).

### 2.3. Statistics

Data analysis was performed using R 4.1.2 software. For data smoothing, the Loess method was used. For data analysis with CRDS (sampling rate = 1 s), mean values were taken for those periods in which the data variation was less than 5% (measurement periods of 20–30 min). The Δ*δ*^13^C-CH_4_ values for both experiments are presented as the arithmetic means of the respective replicates, together with their standard deviations (SD). The arithmetic means and SDs were calculated using Microsoft Excel (Microsoft Excel for Office 365 MSO).

## 3. Results

### 3.1. Oral Intake of Isotopically Labeled DMSO and Measurements of Breath Air

**Figure 3:**
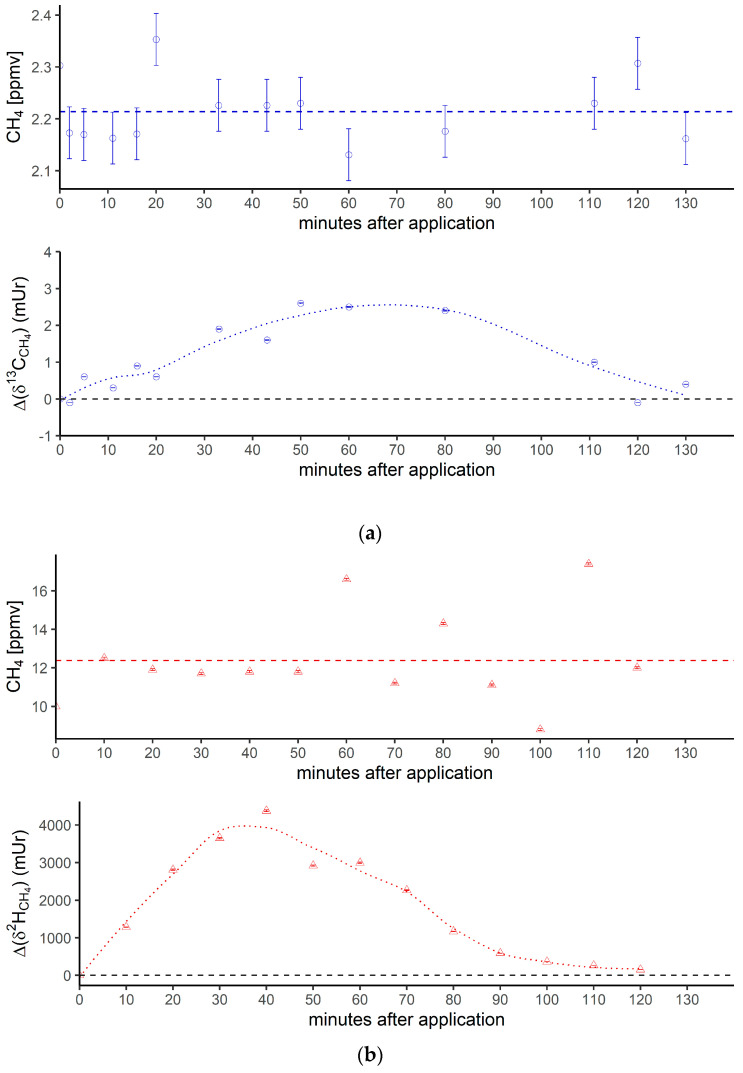

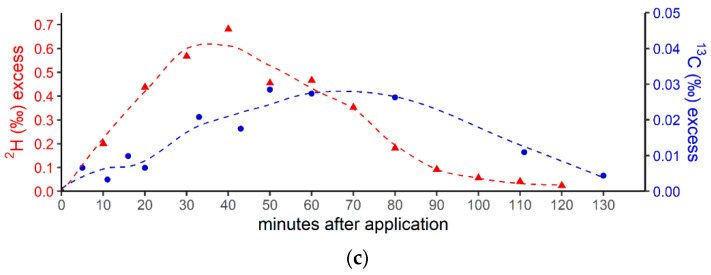
(**a**) Breath CH_4_ production (top) and isotope difference as *δ*^13^C-CH_4_ values relative to control (bottom) after the oral intake of isotopically labeled ^13^C DMSO. The dashed line in the upper figure represents the CH_4_ mean value. Error bars represent a sum of the analytical uncertainties and statistical errors from triplicate measurements. (**b**) Breath CH_4_ production (top) and isotope difference of *δ*^2^H-CH_4_ values relative to the control (bottom) after the oral intake of isotopically labeled ^2^H DMSO. The dashed line in the upper figure represents the CH_4_ mean value. Error bars represent a sum of analytical uncertainties and statistical errors from triplicate measurements, and lie within the symbols. (**c**) ^2^H (red) and ^13^C (blue) isotopic excess after the oral administration of labeled ^13^C and ^2^H DMSO, respectively.

Figure 3 shows the breath CH_4_ production and isotope differences as *δ*^13^C-CH_4_ values (Figure 3a) and *δ*^2^H-CH_4_ values (Figure 3b), relative to the control values, which were monitored using the breath air of the subject after an oral intake of isotopically labeled (with ^13^C and ^2^H, respectively; see the Materials and Methods section) DMSO over 130 min. Breath CH_4_ production (with laboratory background values subtracted) during the monitoring period showed mean values of 2.2 ± 0.06 ppmv (Figure 3a, top) and 12.4 ± 2.3 ppmv (Figure 3b, top), respectively.

At the beginning and end of the experiment (after 130 min) the Δ*δ*^13^C-CH_4_ and Δ*δ*^2^H-CH_4_ values of around 0 mUr closely reflected the average isotopic signature of the volunteer’s breath *δ*^13^C-CH_4_ values and *δ*^2^H-CH_4_ values. For details regarding the determination of the volunteer’s isotope CH_4_ source signatures observed for several periods in 2018 and 2019 without any treatment of isotopically labeled compounds, see the Supplementary Materials. Within a few minutes of the oral intake of isotopic labeled DMSO, the Δ*δ*^13^C-CH_4_ and Δ*δ*^2^H-CH_4_ values substantially increased toward less negative relative *δ*^13^C/^2^H-CH_4_ values, resulting in positive Δ values and becoming clearly distinguishable from background values. Maximum increases in Δ values of around 2.5 mUr and 4000 mUr for carbon and hydrogen, respectively, were found between 60 and 40 min. Afterward, the Δ*δ*^13^C-CH_4_ and Δ*δ*^2^H-CH_4_ values steadily decreased, almost reaching the initial *δ*^13^C/^2^H-CH_4_ values after 130 min.

In addition, Figure 3c compares the excess of the isotopic labels in the released breath CH_4_ from the supplemented ^13^C- and ^2^H-DMSO. The excess in both ^13^C-CH_4_ and ^2^H-CH_4_ gradually increased, with maximum values observed at 40 min for ^2^H-CH_4_ (~0.68‰) and 50 min for ^13^C-CH_4_ (~0.028‰). The calculated time integral (area under the curve) values were 37 and 2.14 for ^2^H-CH_4_- and ^13^C-CH_4_-excess, respectively. Both isotope tracers evidently indicated partial conversion of the methyl group of DMSO to CH_4_ by processes within the human body. The calculated time integral found for ^2^H-CH_4_ was by a factor of around 17 higher when compared with ^13^C-CH_4_. In this context, it should be noted that the amount of applied isotope ^13^C-labeling of DMSO was much lower for the ^13^C experiments (see the Discussion section).

### 3.2. Blood Samples and Addition of Isotopically Labeled DMSO and Methionine

**Figure 4:**
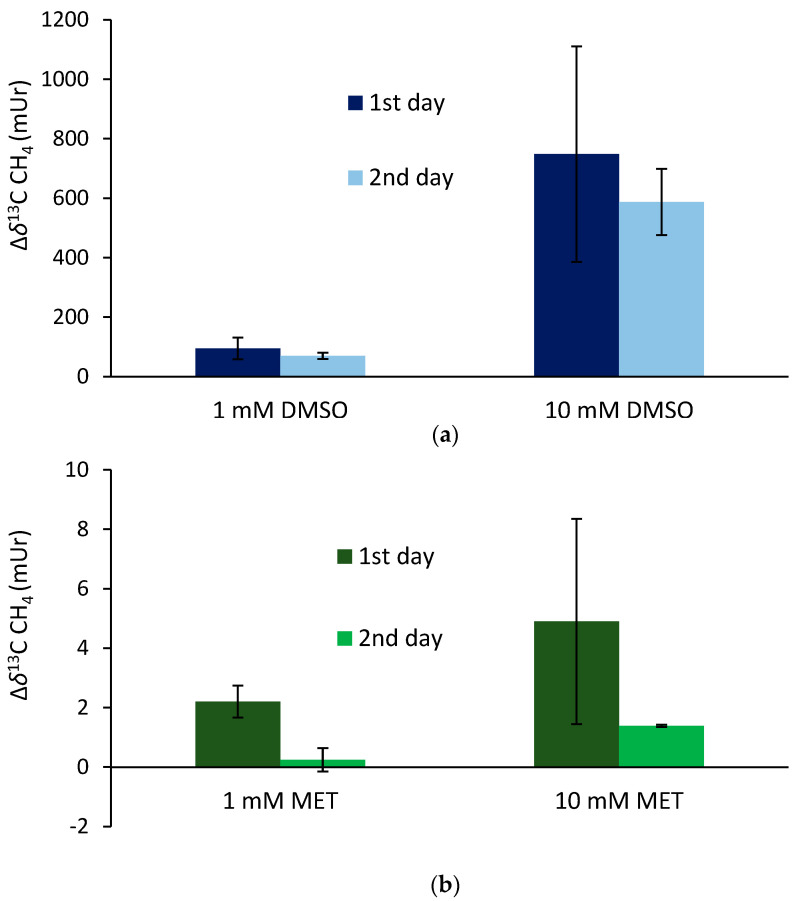
Isotope difference (relative to the control values) of *δ*^13^C-CH_4_ values of headspace air after the treatment of blood samples with ^13^C-labeled DMSO (**a**) and methionine (**b**) incubated over two days at a temperature of 36 °C. Mean values of three replicated experiments (*n* = 3) are shown and error bars mark the SD. Control samples (blood without the addition of isotopically labeled compounds) did not show any measurable difference in *δ*^13^C-CH_4_ values over the incubation time, and thus, are not graphically illustrated.

The supplementation of ^13^C-labeled DMSO and methionine at equimolar concentrations of 1 mM to the blood samples incubated for 24 h (first day) at 36 °C resulted in mean Δ*δ*^13^C-CH_4_ values of 95 ± 36 mUr and 2.2 ± 0.5 mUr for DMSO and methionine, respectively (Figure 4). Repeated measurements of the same samples (after equilibration with laboratory air; see the Materials and Methods section) and another incubation period of 24 h (second day) exhibited lower mean Δ*δ*^13^C-CH_4_ values, producing 70 ± 10 mUr and 0.24 ± 0.4 mUr for DMSO and methionine, respectively. The application of ten-fold higher concentrations of DMSO and methionine (10 mM) enhanced the formation of isotopically labeled CH_4_, with the Δ*δ*^13^C-CH_4_ values producing 748 ± 362 mUr and 4.9 ± 3.5 mUr for DMSO and methionine, respectively. Again, repeated measurements of the same samples after another incubation period of 24 h (second day) exhibited lower mean Δ*δ*^13^C-CH_4_ values, producing 588 ± 10 mUr and 1.4 ± 0.1 mUr for DMSO and methionine, respectively. Thus, the change in 10-fold concentrations was closely reflected by the change in Δ*δ*^13^C-CH_4_ values (factor of ~8) for both days, whilst for methionine, the change in Δ*δ*^13^C-CH_4_ values was lower (factors of 2.2 and 5.7 for day 1 and day 2, respectively). All control samples including blood without the addition of isotopically labeled compounds did not show any measurable difference in *δ*^13^C-CH_4_ values over the incubation time.

### 3.3. Skin Application of Isotopically Labeled DMSO and Incubation of Arm with Exposure to Natural Sunlight

**Figure 5:**
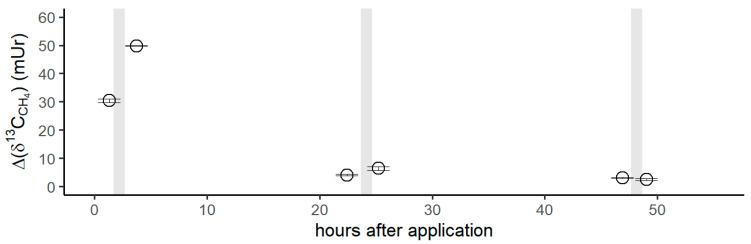
Isotope difference of *δ*^13^C-CH_4_ values (Δδ^13^C-CH_4_) relative to the control values after the application of ^13^C DMSO on the left forearm of the volunteer. Grey vertical lines represent periods of exposure to sunlight.

Figure 5 shows the isotope difference as *δ*^13^C-CH_4_ values relative to the control values after the application of ^13^C-labeled DMSO on the left forearm. Subsequent to the DMSO application, the *δ*^13^C-CH_4_ values increased by 30 mUr within 1 h. After the volunteer exposed his left forearm to natural sunlight in the field, a maximum Δ*δ*^13^C-CH_4_ value of 50 mUr was observed. Please note that direct measurements during exposure to sunlight in the field were not possible. For experimental details, we refer the reader to the Materials and Methods section. After 24 h, *δ*^13^C-CH_4_ values measured for CH_4_ release from the skin of the left forearm were still enriched by 4 mUr, whilst the control values (the incubation of the untreated right forearm) did not show any measurable changes. Again, exposure to sunlight in the field and subsequent laboratory measurements of CH_4_ release from the skin of the forearm increased the Δ*δ*^13^C-CH_4_ value to 6 mUr. After 48 h, the *δ*^13^C-CH_4_ values monitored from the release of the skin still showed a marginal but measurable ^13^C enrichment of 1 mUr. After sunlight irradiation, no measurable increase in *δ*^13^C-CH_4_ values was noted. The associated CH_4_ concentrations of the chamber measurement series showed changes in the range of 1.96 to 2.08 ppmv, which were close to the variations observed for the control measurements.

## 4. Discussion

### 4.1. Conversion of Methylated Sulfur Compounds to Methane

The three sets of experiments—involving the application of two potential CH_4_ precursor compounds, DMSO and methionine, with isotopic labels—provided independent lines of evidence for partial conversion of the supplemented methyl group to CH_4_ in the human body. The combination of the three experiments (oral intake, blood incubations, and skin application) was undertaken to confirm that CH_4_ is endogenously formed in humans via a ROS-driven process without involvement of the well-known microbial sources (methanogens) occurring under anoxic conditions in the gastrointestinal tracts. However, we are aware that it is almost impossible to exclude the contribution of microbes during the screening of humans for CH_4_ emissions.

### 4.2. Oral Administration of ^13^C-Labeled DMSO

**Figure 6:**
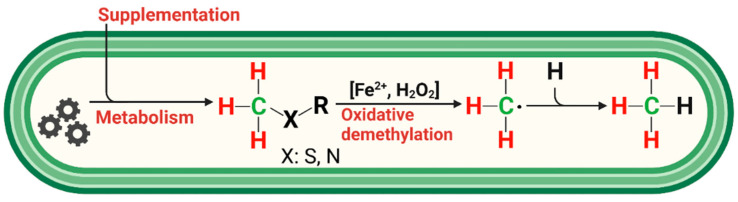
Simplified reaction scheme for endogenous CH_4_ formation in humans. Methylated S-/N-compounds produced via metabolism or externally supplemented act as •OH scavengers or react with oxo-iron(IV) ([Fe^IV^=O]^2+^) to produce methyl radicals. Activation of hydrogen peroxide by ferrous iron (Fenton systems) leads to several oxidizing agents, such as [Fe^IV^=O]^2+^ or hydroxyl radicals, depending on the reaction conditions. Subsequently, CH_4_ is formed through the reaction of a methyl radical with a hydrogen atom derived from hydrocarbons, hydrogen peroxide, or hydrogen carbonate. Red and green indicate hydrogen and carbon atoms, respectively, of methylated sulfur compounds labeled with ^2^H and ^13^C, as applied in this study to subsequently trace the formation of CH_4_ in humans.

The measured isotopic changes for the two labeling experiments (Figure 3) unambiguously demonstrated that the methyl group of DMSO was converted to CH_4_. The ^2^H and ^13^C excess values indicated that only a marginal fraction (0.68‰ and 0.028‰) of the CH_4_ concentration measured in the subject’s breath air (~2 to 16 ppmv) was actually derived from the isotopically labeled precursor methyl groups of DMSO. The observed variabilities in concentrations during the individual experiments (Figure 3a,b, top panels) were in the range of the intraday fluctuations. The observed difference in CH_4_ base levels of approximately 10 ppmv between the experiments with ^13^C DMSO and ^2^H DMSO reflected usual changes in the individual’s breath CH_4_ state, as the two experiments were performed a few months apart. For details regarding the variabilities of CH_4_ base levels of the volunteer, see Polag and Keppler [37,38]. The small concentration changes indicated by the supplementation of ^13^C-labeled DMSO would be nondetectable when using conventional measurement techniques, and can only be traced using isotopic labeling techniques. To better compare the conversion of the two labeling approaches, it is necessary to consider the ^2^H/^13^C excess values, as shown in Figure 3c. The calculated time integrals of the ^2^H-CH_4_- and ^13^C-CH_4_ excesses were 37 and 2.14, respectively, and thus, the time integral found for the ^2^H-CH_4_ excess was higher by a factor of around 17 when compared with the ^13^C-CH_4_ excess. Please note that the ^2^H-CH_4_ excess time integral of 37 included three deuterium atoms from a ^2^H-labeled methyl group and a fourth, unlabeled hydrogen atom (see Figure 6). To correct for this effect, the time integral of ^2^H increased to 49, and the differences between the excess values of ^2^H-CH_4_ and ^13^C-CH_4_ changed to a factor of 23. This value closely reflected the relationship between orally administered ^2^H and ^13^C isotope tracers (factor of 34). The reason for applying different amounts of ^2^H/^13^C DMSO isotopic labels was due to financial issues, as ^2^H-labeled DMSO is considerably cheaper than ^13^C-labeled DMSO. Nevertheless, both isotope tracers independently and clearly indicated similar conversion rates of the methyl group of DMSO when normalized to the amount of applied isotopic tracer. We suggest that the observed CH_4_ formation is indicative of the formation of methyl radicals from DMSO induced by hydroxyl radicals or oxo-iron(IV) species, as recently proposed by Ernst et al. [17], Benzing et al. [52], and Althoff et al. [51] for biological and abiotic chemical systems. Once methyl radicals are formed, they can react with a hydrogen atom from hydrocarbons, hydrogen peroxide, or hydrogen carbonate to form CH_4_. The formation of ^13^C-enriched CH_4_ was already measurable a few minutes after the oral intake of the labeled substance for both isotope labeling experiments (^2^H and ^13^C). However, around 2 h after the oral administration, CH_4_ formation from DMSO was barely detectable in the breath air, potentially implying that most of the DMSO was converted in the human body within this timespan. A possible decay mechanism is the conversion of DMSO to dimethyl sulfide (DMS) by the molybdoenzyme DMSO reductase, which is widespread in all domains of life [69]. A recently proposed mechanism of DMSO reductase can be found in Le et al. [70].

### 4.3. Supplementation of ^13^C-Labeled DMSO and Methionine to Blood Samples

The experiments with blood samples were conducted to further demonstrate the non-microbial formation of CH_4_ when different S-methylated compounds were supplemented. When equimolar amounts of DMSO and methionine were added to the blood samples, the conversion of S-methyl-bonded groups to CH_4_ was much higher for DMSO than for methionine, with factors ranging from 43 to 423. It is well known that DMSO is a potent hydroxyl radical scavenger [71] that forms CH_4_; ethane; and oxidized C1 compounds, such as formaldehyde and formate, depending on the experimental conditions [72,73,74]. The observed differences between the application of DMSO and methionine are in line with previous experiments conducted by Althoff et al. [51] and Ernst et al. [17], who showed the preferential formation of ROS-induced formation of CH_4_ from DMSO relative to methionine in chemical systems and living organisms, respectively. However, in our study, the difference between DMSO and methionine was even more pronounced and might be explained by the specific composition of the blood samples, i.e., amounts and availability of iron species and ROS. In addition, methionine needs to be oxidized to methionine sulfoxide before the methyl groups can be cleaved off [51]. Human blood and plasma contain high amounts of iron species, particularly in the form of hemoglobin, and the range of H_2_O_2_ might be in a normal concentration range of 1–5 µM but increases to 30–50 µM during chronic inflammation in certain disease states [75]. Thus, the interplay between iron species and ROS in blood might be highly supportive for the formation of CH_4_ given that the required methyl precursor compounds are also available. Interestingly, a ten-fold higher DMSO supplementation was well reflected by the amounts of formed labeled CH_4_ (factor of ~8), whilst a considerably lower increase was observed (mean factor of ~4) for the addition of methionine. It was also obvious that CH_4_ formation from DMSO was observable for much longer (at least for 48 h) in the blood samples when compared with the oral administration of DMSO (see section above), indicating that different degradation processes in the human body might have contributed to the observed pattern.

### 4.4. Dermal CH_4_ Emissions after Treatment of Isotopically Labeled DMSO

The application of ^13^C-labeled DMSO on the volunteer’s forearm clearly showed the release of isotopically labeled CH_4_ immediately after incubation of the penetrated skin section (Figure 5) under laboratory conditions. Based on our current understanding—including knowledge of ROS-driven CH_4_ formation, and that DMSO rapidly penetrates through human skin—this observation is highly indicative of methyl radical formation induced by ROS that occurs in the epidermis or dermis of the skin. There is frequent formation of ROS in the cells and it is well known that skin exposure to light—including wavelengths of visible, UVA/UVB, and IR light —induces and increases ROS levels [76,77]. After the volunteer exposed his left forearm to natural sunlight in the field for 1 h, a strong isotope change in *δ*^13^C-CH_4_ values (~70% higher relative to laboratory light exposure) was measured, even though these measurements were conducted after exposure to direct solar radiation. This implies that enhanced levels of ROS were caused by the irradiation of solar light, leading to the formation of CH_4_, which could only be made visible by the administration of ^13^C-labeled DMSO. After around 24 h, the release of ^13^C-labeled CH_4_ from the skin under laboratory incubation conditions was still measurable and increased again (by about 50%) after the exposure of the skin to natural sunlight. When repeating the same procedure after 48 h, a small but indicative change in *δ*^13^C-CH_4_ values was still observed for the laboratory exposure incubations of the forearm. No additional increase in *δ*^13^C-CH_4_ values could be measured for the effect of natural sunlight. However, it was remarkable to observe DMSO-related liberation of CH_4_ from the skin even 50 h after the application of ^13^C-labeled DMSO. There exist only a few studies that dealt in detail with the release of CH_4_ from human skin, and in general, these emissions are considered to be much smaller than those measured for breath release [35]. This was recently confirmed by Li et al. [78], who quantified dermal and exhaled CH_4_ of 20 volunteers using climate chambers and reported that the average estimated exhaled CH_4_ release rate was about 19 (max. range 13–37) times higher than the average dermal CH_4_ emission rate. For completeness, it should be noted that Mochalski et al. [79] measured emission rates of selected volatile organic compounds from the skin of healthy volunteers. However, the researchers did not detect CH_4_, as they screened for larger carbon compounds, including C4 to C10 substances, and found relatively large emissions for three volatiles: acetone, acetaldehyde, and 6-methyl-5-hepten-2-one.

### 4.5. ROS-Induced non-Microbial Formation of CH_4_ from Methylated S-/N-Compounds in Humans: A Hypothesis

The observed formation of CH_4_ from the S-bonded methyl groups of DMSO or methionine provides strong support for a radical-driven process of CH_4_ formation. Based on the three applied isotopic labeling experiments and a previous study demonstrating ROS-driven CH_4_ formation from in vitro experiments of many organisms [17], we propose a reaction scheme showing the interplay of methyl precursors, ROS, and iron species that eventually leads to the formation of CH_4_ in humans (Figure 6).

The three major players in this reaction scheme are ROS, iron, and methyl groups bonded to sulfur and nitrogen compounds. Below, we briefly summarize their role in humans with respect to non-microbial CH_4_ formation.

Initially considered principally toxic, today, ROS are well-known for having beneficial or deleterious effects in aerobic organisms [59,80,81,82]. The concentration of H_2_O_2_ in the normal cytoplasm, mitochondrial matrix, and endoplasmic reticulum (ER) lumen varies by several orders of magnitude (from 80 pM to 700 nM) [83] and is even higher in blood and plasma at normal concentrations of 1–5 µM, but increases to 30–50 µM during chronic inflammation in certain disease states [75]. On the one hand, ROS play various roles in the cellular functioning of aerobic organisms, are involved in many redox-governing activities of the cells for the preservation of cellular homeostasis, and are required for many important signaling reactions. On the other hand, elevated ROS levels can lead to severe damage in cells. In this context, it was suggested that frequently increased oxidative stress leads to an overproduction of ROS, causing many diseases and a variety of age-related disorders, such as Parkinson’s disease, Alzheimer’s dementia, chronic inflammatory diseases, atherosclerosis, heart attacks, cancer, ischaemia/reperfusion injury, and arteriosclerosis. Thus, it can be easily envisaged that CH_4_ might be formed at highly fluctuating levels in different organelles and might potentially serve to monitor enhanced ROS levels in humans. This hypothesis is supported by the results of several recent monitoring studies:

(I) The observation that breath CH_4_ levels increase with advanced age [24] might be an indication of the human age-related increase in systemic inflammation accompanied by enhanced ROS levels. (II) Long-term monitoring studies of breath CH_4_ from several volunteers provided evidence that abrupt deviations in breath CH_4_ levels from baseline were linked to inflammatory processes and immune reactions [37]. In this context, infectious diseases were mostly accompanied by temporarily elevated breath CH_4_ formation. Next, it was hypothesized that vaccinations as induced perturbations of the immune system might cause substantial fluctuations in the breath CH_4_ level of people, indicating individual immune responses and immune states. (III) This was recently shown by Polag and Keppler [38], who investigated the breath CH_4_ levels after COVID-19 vaccination. They clearly found large deviations from the average breath CH_4_ values after vaccination and concluded that these deviations were likely related to immune reactions and may have also originated from redox homeostasis in cells. A change in the breath CH_4_ levels from individual baseline values could be used to monitor changes in levels of ROS and oxidative stress, and could potentially be used to classify immune responses. (IV) Finally, Tuboly et al. [84] investigated the possibility of CH_4_ generation in low-CH_4_ emitters that consumed high doses of ethanol with the aim to increase oxidative stress. A transient, significant CH_4_ production was noted after an excessive ethanol intake. The researchers found similar results when they repeated the ethanol experiments with rats. They further investigated the hypothesis that L-alpha-glycerylphosphorylcholine (GPC) may influence CH_4_ formation through the modulation of alcohol-induced mitochondrial dysfunction.

This brings us to the next point: to counteract oxidative stress, aerobic cells possess many antioxidative systems that function to keep the ROS level in a non-toxic range. Methyl precursors—particularly those where the methyl group is bonded to sulfur and nitrogen compounds—can readily be cleaved off to produce CH_4_ or oxidized C1 species [17,51,52]. The various available S-/N-methylated compounds in biological systems will cause different efficiencies of CH_4_ production and consumption of ROS. DMSO is not produced in humans and is only consumed via the diet in relatively small quantities [85]. However, this effective radical scavenger was ideally suited to test the hypothesis of non-microbial CH_4_ formation in humans. It is non-toxic in the applied doses, penetrates rapidly through human skin, and is easily distributed in the body, as it dissolves in both polar and nonpolar compounds. On the other hand, the other applied S-methylated compound, namely, methionine, is an essential amino acid in humans that has an important role in metabolism and health. It is the precursor of other important compounds, such as cysteine, S-adenosyl methionine (SAM), and glutathione. It was also shown to produce CH_4_, albeit at much lower conversion rates when compared with DMSO.

Nitrogen-methylated substances, such as choline (2-Hydroxyethyl-trimethylammonium), are formed in humans but are also essential compounds for maintaining health. Therefore, they must be consumed by diet as choline or as choline phospholipids. Large amounts of choline are stored in the human cell membranes and organelles as phospholipids, and inside cells as phosphatidylcholines and GPC. Choline was shown to form CH_4_ in a chemical model system containing iron and hydrogen peroxide [51,86] but this was not confirmed in bacterial culture experiments [17]. Tuboly et al. [84] showed that exogenous GPC protected against ethanol-induced mitochondrial electron transport chain dysfunction in rat liver, which is the primary target of alcohol-induced oxido-reductive stress. Therefore, the exogenous addition of methylated compounds might strongly increase CH_4_ production and ROS consumption. In this context, it is of interest to further discuss the potential role of DMSO as an effective scavenger of radicals to counteract enhanced oxidative stress induced by ROS. DMSO has already been investigated for many years, but its beneficial role for medical use remains highly uncertain (see the Introduction section).

Finally, the concentration of free iron (in the form of iron(II)) is of importance for the enhanced production of hydroxyl radicals (Fenton-type reactions) in biological systems [59]. However, inappropriately low or high levels of iron are detrimental and contribute to a wide range of diseases [87]; therefore, understanding the dysregulation of iron metabolism is crucial in the search for therapeutics [88]. Harmful oxidative distress could be observed in states of both iron deficiency (anemia) and overload (ferroptosis) [89]. It is plausible that appropriate supplementation of iron is beneficial to health, which may be related to its role in contributing to the homeostasis of cellular ROS through the production of CH_4_.

A detailed understanding of the interplay between ROS, iron, and methylated substrates in humans is necessary to better understand radical-driven CH_4_ and to answer the question of whether the cellular formation of CH_4_ has a physiological role in humans. In this context, monitoring CH_4_ as an indicator for ROS-driven processes could be a promising approach in biochemical research, where breath CH_4_ could be used as a diagnostic tool in the fields of system biology and precision medicine. This could include the application of isotopic labeling experiments of methylated precursor substances (with a ^13^C or ^2^H label), as this approach could specifically visualize ROS-related CH_4_ generation, and thus, overcome the problem of higher breath CH_4_ background concentrations derived from microbial sources. These changes may be interesting for diagnostic purposes. Moreover, the possibility exists that such changes may affect the overall cellular response to intracellular hypoxia. Simple asphyxiants, such as CH_4_, act by physically limiting the utilization of oxygen and can modify the symbiosis with other gaseous compounds within the internal milieu of aerobic cells. Although CH_4_ is conventionally believed to be physiologically inert, a comprehensive view of its biological effects in various hypoxic and inflammatory scenarios was demonstrated [90]. Notably, it was shown that CH_4_ can modulate the pathways involved in key events of inflammation via master switches, such as Nrf2/Keap1 and NF-κB (for a review, see [18]). Several studies also demonstrated that exogenous CH_4_ modulates the intrinsic, mitochondrial pathway of pro-apoptotic activation in model experiments [91]. Furthermore, sequential in vitro studies with exogenous normoxic CH_4_ in simulated ischemia-reperfusion environments provided evidence that CH_4_ preserves the mitochondrial respiratory capacity in cells exposed to anoxia [92]. In a similar protocol, CH_4_ treatment restricted the forward electron transfer within complex I in control mitochondria while effectively restricting reverse electron transport (RET) in post-anoxic mitochondria. In parallel studies, CH_4_ influenced several components of the endoplasmic reticulum-mitochondria-related pro-apoptotic signaling pathways, the oxidative phosphorylation capacity was more preserved, and the relative mRNA expression for hypoxia- and ER stress-associated genes (including HIF-1α) was significantly reduced [93]. For a detailed discussion regarding the potential applications of monitoring CH_4_ in medical research and health sciences, see [18,38,94,95].

## 5. Conclusions

We are aware that the investigation of ROS-driven cellular CH_4_ formation in only one subject is too low of a sample size to draw broad and general conclusions. However, this study represents the first proof of concept that cellular CH_4_ formation occurs in the human body and is most likely a result of the interplay between ROS and methylated substrates. This process can currently only be made clearly visible by applying stable-isotope-tracing techniques to distinguish CH_4_ formation in humans from that of microbes living in the gastrointestinal tract. Together with other recently published studies [17,38,94], it is becoming obvious that ROS-driven CH_4_ formation might be a necessary phenomenon of aerobic life. Consequently, non-microbial aerobic CH_4_ formation should be highly variable in time and source strength, as it may be an integral part of the cellular responses toward changes in oxidative status present in humans. Large changes in human breath levels were observed by several recent monitoring studies [38,78,84], and some suggested that variations in CH_4_ breath levels are unlikely to be explained by microbial formation in the human digestive system. However, additional investigations are required to obtain unambiguous evidence of non-microbial CH_4_ formation in humans and the underlying processes of its generation. This will be a significant challenge because in the case of high emitters—where CH_4_ formation by methanogens is the dominant process—it is difficult to distinguish between the non-microbial and microbial pathways of CH_4_ production. Nevertheless, for low and medium CH_4_ emitters, who comprise about 70% of the global population, we suggest that substantially changed human breath CH_4_ levels from individual baseline values may be used to detect changes in oxidative stress and ROS levels, and could potentially be used to classify immune responses, as recently suggested by Polag and Keppler [38]. Therefore, future investigations should focus on deciphering the potential physiological role of CH_4_ formation in humans, as well as on the monitoring of CH_4_ as an indicator for individual immune states and a potential biomarker of oxidative stress. In addition, revisiting and studying in greater detail the potential role of DMSO as an effective hydroxyl radical scavenger and its use for human medical research might be worthwhile.

## Data Availability

The data used in this publication are available to the community and can be accessed by request to the corresponding author.

## Supplementary Materials

The Supplementary Materials for this article can be downloaded from https://www.mdpi.com/article/10.3390/antiox12071381/s1. Figures S1 and S2: Determination of stable isotope source signatures of CH_4_ using keeling plots: Photo S1: Arm incubation chamber for online measurements using CRDS; Table S1: Overview and timeline of isotope labeling experiments.

## Abbreviations

AscariteII^®^	sodium-hydroxide-coated silica
C	carbon
CH_4_	methane
•CH_3_	methyl radicals
CO_3_*^–•^*	carbonate radicals
CO_2_	carbon dioxide
CRDS	cavity ringdown spectroscopy
D	deuterium
DMSO	dimethyl sulfoxide
Drierite^®^	anhydrous calcium sulfate
ER	endoplasmatic reticulum
EDTA	Ethylenediaminetetraacetic acid
FDA	Food and Drug Administration
[FeIV=O]^2+^	nonheme oxo-iron(IV)
Fe^2+^	ferrous iron
Fe^3+^	ferric iron
FID	flame ionization detector
GC	gas chromatography
GPC	L-alpha-glycerylphosphorylcholine
H_2_	hydrogen
H_2_O_2_	hydrogen peroxide
IAEA	International Atomic Energy Agency
IRMS	isotopic ratio mass spectrometry
N	nitrogen
NIST	National Institute of Standards and Technology
•OH	hydroxyl radicals
O_2_*^–•^*	superoxide radicals
PI	principal investigator
ppvb	parts per billion by volume
ppmv	parts per million by volume
PTFE	polytetrafluorethylene
RET	reverse electron transport
ROS	reactive oxygen species
S	sulfur
SAM	S-adenosyl methionine
SD	standard deviation
SI	system of units
TC	thermal conversion
mUr	milliurey
V-PDB	Vienna Pee Dee Belemnite
V-SMOW	Vienna Standard Mean Ocean Water
