# Supplementary material for: Radical-Driven Methane Formation in Humans Evidenced by Exogenous Isotope-Labeled DMSO and Methionine
Source: Antioxidants (Basel). 2023 Jul 4;12(7):1381. doi: 10.3390/antiox12071381 (PMC10376501; doi:10.3390/antiox12071381)

Supplementary Material

Frank Keppler[1,2]*, Mihály Boros[3], Daniela Polag[1]
[1]Institute of Earth Sciences, Heidelberg University, D-69120 Heidelberg, Germany
[2]Heidelberg Center for the Environment (HCE), Heidelberg University, D-69120 Heidelberg, Germany
[3]Institute of Surgical Research, University of Szeged, H-6724 Szeged, Hungary
*Correspondence: frank.keppler@geow.uni-heidelberg.de

Time frame of experiments

Table S1: Overview and timeline of isotope labeling experiments.

experimental series	date
oral intake ^{13}C labeled DMSO	06/14/2018
oral intake ^{2}H labeled DMSO	10/12/2020 – 10/13/2020
arm incubation	07/17/2018 – 07/19/2018
blood experiments	07/03/2019 – 07/05/2019

Determination of stable isotope source signatures of CH_4 using keeling plots

The Keeling plot was first used to estimate the $\delta^{13}C$ value of respired CO_2 in an ecosystem from night-time air samples. The basis of the Keeling plot method is conservation of mass. Thus, in our case the concentration of CH_4 in breath air and adjacent atmospheric air (inhaled air) reflects the combination of background atmospheric concentration and the variable amounts of that gas added by sources in the human body.

$$c_a = c_b + c_s \quad (S1)$$

where c_a, c_b, and c_s are, respectively, the breath (exhaled air) CH_4 concentration measured from the volunteer (Fig. S1A), the background CH_4 concentration (inhaled air) and the additional

concentration component produced in the human body which has raised atmospheric CH_4 concentration above background.

Given conservation of mass,

$$\delta^{13}C_a c_a = \delta^{13}C_b c_b + \delta^{13}C_s c_s \quad (S2)$$

where $\delta^{13}C$ represents the stable carbon isotope value of each CH_4 component. Combining equations (S1) and (S2),

$$\delta^{13}C_a = c_b(\delta^{13}C_b - \delta^{13}C_s)\,(1/c_a) + \delta^{13}C_s \quad (S3)$$

where $\delta^{13}C_s$ is the integrated value of the CH_4 sources in the human body. This is illustrated graphically in Figure S1 with breath samples from the volunteer that were collected over a period of four weeks.

The extrapolated intercept of the straight line in the Keeling plot corresponds to the situation when the concentration is very large and dominated by sample CH_4 ($1/[CH_4] = 0$) and thus provides the isotope ratio of the breath CH_4 source. The Keeling plot of isotopic composition versus inverse concentration $1/[CH_4]$ as shown for the volunteer in Figure S1B, yielded a very good linear correlation ($R^2 > 0.96$) and the $\delta^{13}C$ source signature of –76 ± 0.93 mUr was derived by extrapolating to $CH_{4\rightarrow\infty}$ which corresponds to the y-axis intercept of the linear fit to the $\delta^{13}C$ values versus $1/[CH_4]$ data.

The same principle as explained before for stable carbon isotopes was also applied for determining the source signature of stable hydrogen isotopes of breath CH_4 and a δ^2H source signature of –356 ± 5.5 mUr was calculated (Fig. S2).

It is important to recognize that the model described by equations (S1) to (S3) involves two basic assumptions. Firstly, we assume that a simple mixing of only two gas components is considered (a source and the bulk background). Secondly, we assume that the isotope ratio of these two components does not change over the course of the observation.

(A)

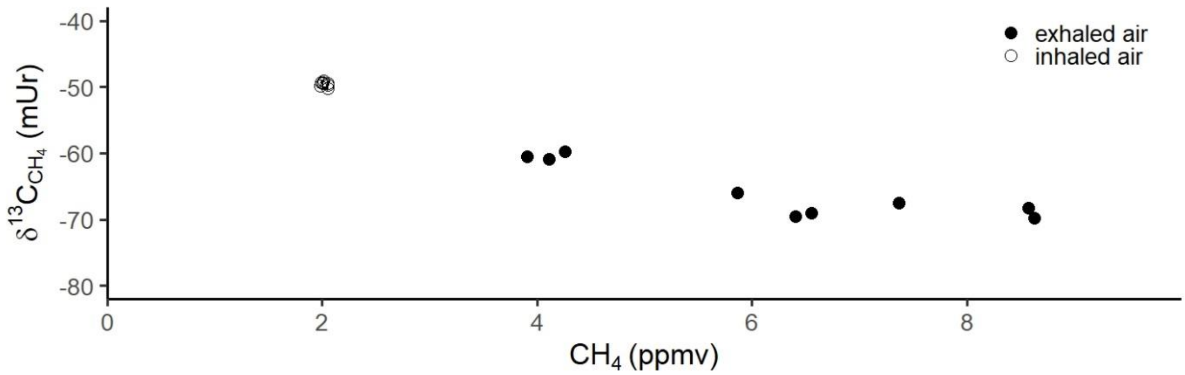

(B)

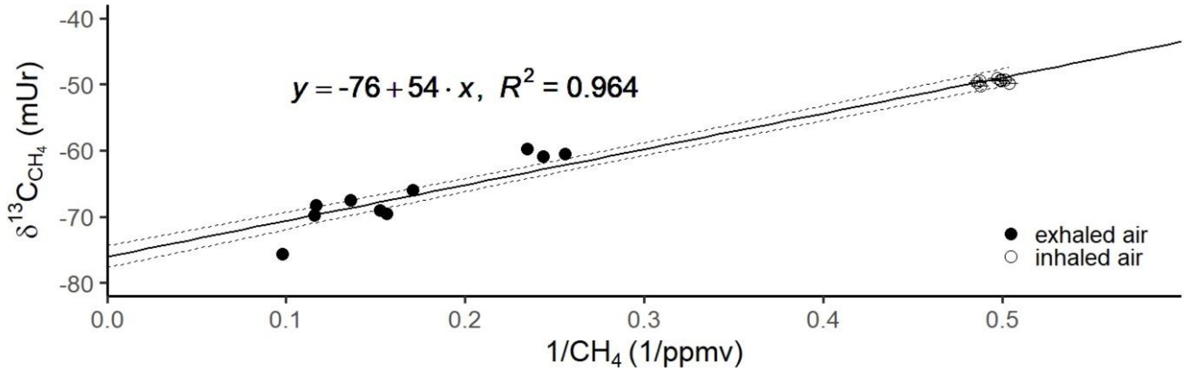

Supplementary Figure S1: (A) Relationship between breath CH_4 concentration and stable carbon isotope values of the subject of breath samples collected within the period of July to August 2019. **(B)** The keeling plot shows the linear regression between 1/CH_4 and $\delta^{13}C_{CH4}$ values including the 95% confidence band of the regression line. The standard errors for the linear regression are 0.93 mUr for the intercept and 2.6 mUr for the slope.

(A)

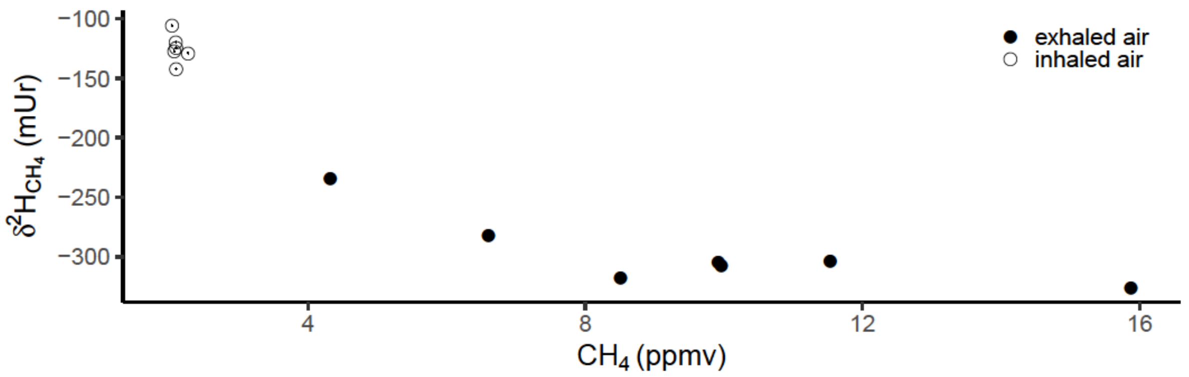

(B)

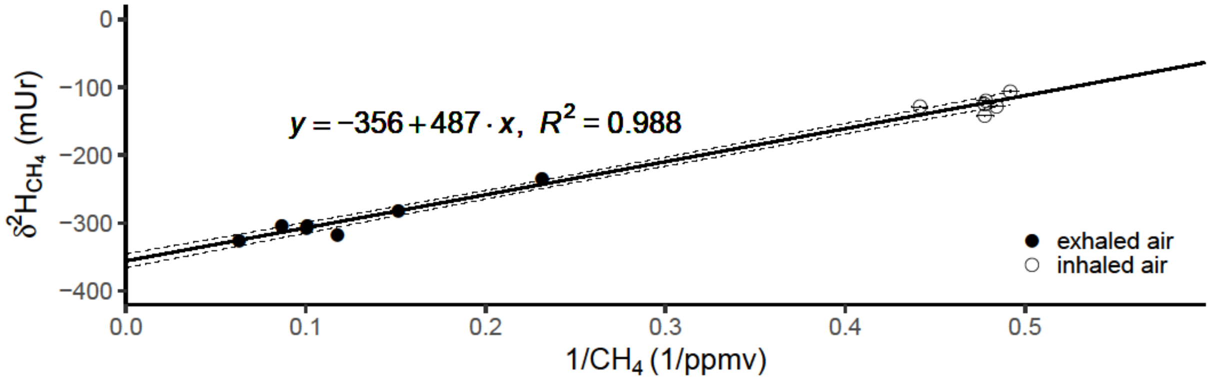

Supplementary Figure S2: (A) Relationship between breath CH_4 concentration and stable hydrogen isotope values of the subject of breath samples collected within the period of July to August 2019. **(B)** The keeling plot shows the linear regression between 1/CH_4 and δ^2H_{CH4} including the 95% confidence band of the regression line. The standard errors for the linear regression are 5.5 mUr for the intercept and 16.2 mUr for the slope.

Supplementary photo S1: Arm incubation chamber for online measurements using CRDS

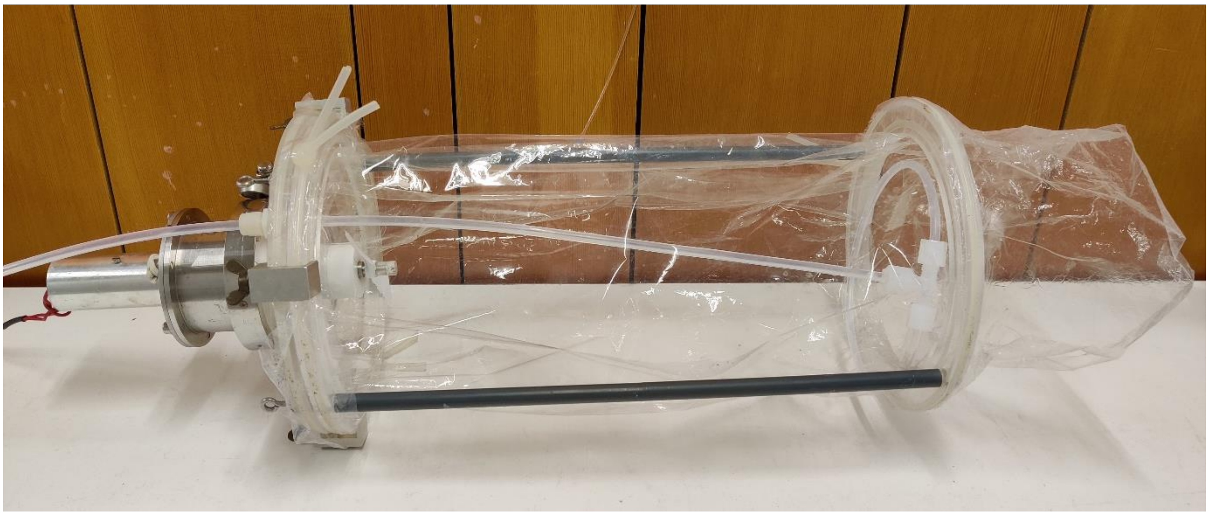

Supplement: Supplementary file 1 [file antioxidants-12-01381-s001.zip › antioxidants-2445244-supplementary.pdf]
